# DFT study on some polythiophenes containing benzo[*d*]thiazole and benzo[*d*]oxazole: structure and band gap

**DOI:** 10.1080/15685551.2021.1971376

**Published:** 2021-09-06

**Authors:** Trung Vu Quoc, Dai Do Ba, Duong Tran Thi Thuy, Linh Nguyen Ngoc, Chinh Nguyen Thuy, Huong Vu Thi, Linh Duong Khanh, Oanh Doan Thi Yen, Hoang Thai, Van Cao Long, Stefan Talu, Dung Nguyen Trong

**Affiliations:** a Faculty of Chemistry, Hanoi National University of Education, Cau Giay, Hanoi; b Nguyen Trai High School, Ba Dinh, Hanoi, Vietnam; c Bien Hoa Gifted High School, Phu Ly City, Ha Nam Province, Vietnam; d Faculty of Training Bachelor of Practice, Thanh Do University, Kim Chung, Hoai Duc, Hanoi Vietnam; e Institute for Tropical Technology, Vietnam Academy of Science and Technology, Cau Giay, Hanoi, Vietnam; f Graduate University of Science and Technology, Vietnam Academy of Science and Technology, Cau Giay, Hanoi, Vietnam; g Publishing House for Science and Technology, Vietnam Academy of Science and Technology, Cau Giay, Hanoi, Vietnam; h Institute of Physics, University of Zielona Góra, Zielona Góra, Poland; i Technical University of Cluj-Napoca, The Directorate of Research, Development and Innovation Management (DMCDI), Cluj county, Romania; j Faculty of Physics, Hanoi National University of Education, Cau Giay, Hanoi, Vietnam

**Keywords:** Polythiophene, band gap, DFT, benzo[*d*]thiazole, benzo[*d*]oxazole

## Abstract

The content of this paper focuses/shed light on the effects of X (X = S in P1 and X = O in P2) in C_11_H_7_NSX and R (R = H in P3, R = OCH_3_ in P4, and R = Cl in P5) in C_18_H_9_ON_2_S_2_-R on structural features and band gaps of the polythiophenes containing benzo[*d*]thiazole and benzo[*d*]oxazole by the Density Function Theory (DFT) method/calculation. The structural features including the electronic structure lattice constant (a), shape, total energy (E_tot_) per cell, and link length (r), are measured via band gap (E_g_) prediction with the package of country density (PDOS) and total country density (DOS) of material studio software. The results obtained showed that the link angle and the link length between atoms were not changed significantly while the E_tot_ was decreased from E_tot_ = – 1904 eV (in P1) to E_tot_ = – 2548 eV (in P2) when replacing O with S; and the E_tot_ of P3 was decreased from E_tot_ = – 3348 eV (in P3) when replacing OCH_3_, Cl on H of P3 corresponding to E_tot_ = – 3575 eV (P4), – 4264 eV (P5). Similarly, when replacing O in P1 with – S to form P2, the E_g_ of P1 was dropped from E_g_ = 0.621 eV to E_g_ = 0.239 eV for P2. The E_g_ of P3, P4, and P5 is E_g_ = 0.006 eV, 0.064 eV, and 0.0645 eV, respectively. When a benzo[*d*]thiazole was added in P1 (changing into P3), the E_g_ was extremely strongly decreased, nearly 100 times (from E_g_ = 0.621 eV to E_g_ = 0.006 eV). The obtained results serve as a basis for future experimental work and used to fabricate smart electronic device.

## Graphical abstract

**Figure:**
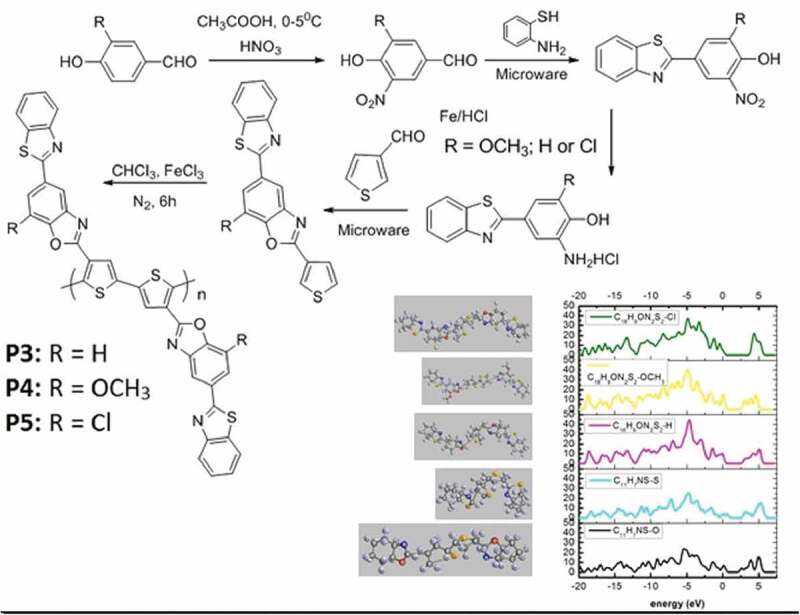

## 1. Introduction

In recent years, polythiophene-containing heterocycles have many advanced applications based on their high environmental sustainability, structural flexibility, optical stability, and electrochemical characteristics [1–12]. They were reported as potential functional materials, such as organic field-effect transistors [13,14], organic light-emitting diodes [15,16], organic photovoltaic cells [17], and other optoelectronic devices [18,19]. Moreover, they also have many applications in pharmacology as water-soluble sensing agents for the recognition of DNA, proteins, and metal ions [20–22], thermochromism, photochromism, and biochemist [23–25]. Among those, benzothiazole-based polythiophenes have attracted much attention thanks to their wide range of biological activities [26,27]. A novel conducting poly[3-(benzothiazole-2-yl)] thiophene polymerized by electrochemical and chemical synthesis has been studied for its optical absorption and photoluminescence characteristics [28–30]. Some technologically advanced methods were applied for the synthesis of 3-(benzothiazole-2-yl)thiophene from the reaction of thiophene-3-carbaldehyde with *o*-amino thiophenol in refluxing ethanol [31] or under microwave radiation without solvent and catalyst [32]. However, very few studies based on the molecular orbital calculations have been performed for oligothiophenes containing heteroaromatic side chains. Basing on theoretical prediction, Radhakrishnan S. *et al*. using suggested the structure optical properties relationship of oligothiophenes having four thiophene units [27,33]. In general, the periodic calculations of geometrical stability and electrical properties of the polythiophenes are often unattended with their published experimental data while these theoretical properties are important bases for deeply understanding the nature and application ability in the energy industry and electrically conducting materials. Therefore, structural analysis based on the theoretical calculation of polythiophene derivatives is very necessary. Among theoretical quantum mechanical methods, Density Functional Theory (DFT) method is well-known as an effective method for evaluation of the transition temperature, electronic properties, and structural characteristics of the *π*-conjugated polythiophene derivatives [34–42]. However, only a few studies on the electronic structures of polymers using the DFT method have been done to control the band gap for determining the alternation between conductors and insulators in solar cells, diodes, or transistors. A popular pathway to synthesize new polythiophene derivatives is substitution in polythiophenes, for example, replacement of S atom with Se or Te atoms [43] or replacement of H atoms with CH_3_, NH_2_, NO_2_, or Cl [44]. The DFT method has been applied for the assessment of structural and electronic characteristics of 4 *H*-cyclopenta[2,1-*b*,3;4-*b′*] dithiophene S-oxide derivatives including X (X: O, S, S = O, BH_2_, SiH_2_) as a bridge [45]. The other five-membered ring molecules and ionization energies (IEs) and the heats of formation of thiophene were calculated and reached a high precision level of ab initio predictions [46]. Recently, the team members also studied the factors affecting the structural, mechanical, and magnetic properties of metal Fe [47,48], Al [49–51], Ni [52–54], Ag [55], alloys AlNi [56], NiCu [57,58], FeNi [59,60], AgAu [61], NiAu [62], and replace the H derivatives of poly C_13_H_8_OS-H with metal atoms Br, Cu, Kr, Ge, As, Fe [63] showed that the E_g_ band gap decreased, leading to an increase in the conductivity. The obtained results will contribute to research to find materials new for application in the industrial age. In addition, the electronic and optical characteristics of 4 *H*-cyclopenta[2,1-*b*:3,4-*b*′]bithiophene derivatives combining with a variety of functional groups including carbon atoms and heteroatoms in the 4-position were predicted using DFT calculations [64]. Along with that, the energy band gap (E_g_) of C_13_H_8_OS was decreased to E_g_ =** **1.621 eV when doping with Br, while the energy band gaps of C_13_H_8_OS was increased to E_g_ = 1.646, 1.697, 2.04, and 1.920 eV, respectively, when doping with H, OH, OC_2_H_5_, or OCH_3_ groups. The obtained results proved that the substituents had a remarkable effect on the link length as well as band gap of polythiophene derivatives, and molecular shape. More recently, our research group studied some novel polythiophenes containing benzo[*d*]thiazole that presented a catalyst- and solvent-free microwave-assisted synthesis of mono thiophene [8]. Their structure and properties were determined by FT-IR, ^1^H-NMR, ^13^C-NMR spectra, single-crystal X-ray diffraction, and the TGA method [32]. We have also mentioned the chemical polymerization of the monomers using anhydrous FeCl_3_ as an oxidant in anhydrous chloroform. However, the structures, phase transition temperature, and electronic property data of these polymers are rather limited. The purpose of this work is to predict the theoretical structure and properties in the ideal state of some newly synthesizing polythiophenes containing benzo[*d*]thiazole and benzo[*d*]oxazole using DFT calculation. With the available results, we are looking forward to synthesize these polythiophenes using chemical polymerization to environmental stability, improve their processability, and electrical properties.

## 2. Computational methods

**Figure 1.**
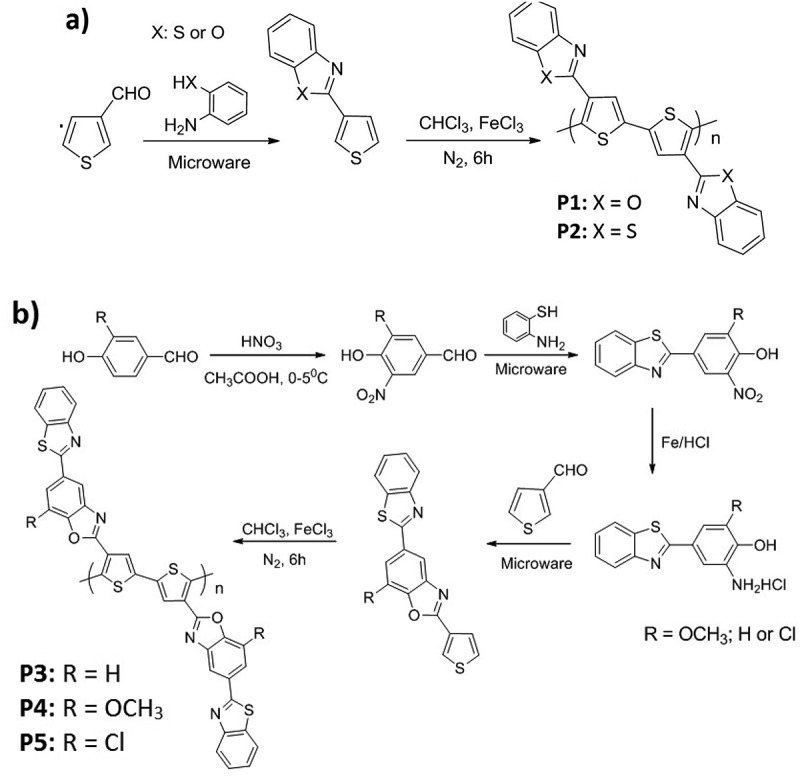
The synthetic procedure of the five novel polythiophenes containing benzo[*d*]thiazole and benzo[*d*]oxazole

The five polythiophenes containing benzo[*d*] thiazole and benzo[*d*]oxazole have been formulated and synthesized according to the process of Material Studio software, such as: Polymer 1 is poly[3-(benzo[*d*]thiazole-2-yl)thiophene], P1: C_11_H_7_NS-O; Polymer 2 is poly [3-(benzo[*d*] oxazole-2-yl)thiophene], P2: C_11_H_7_NS-S; Polymer 3 is poly-3-(5-(benzo[*d*] thiazole-2-yl)-7-methoxybenzo[*d*]oxazole-2-yl)thiophene, P3: C_18_H_9_ON_2_S_2_-H; Polymer 4 is poly-3-(5-(benzo[d]thiazole-2-yl)benzo[*d*]oxazole-2-yl)thiophene, P4: C_18_H_9_ON_2_S_2_-OCH_3_; Polymer 5 is poly-3-(5-(benzo[*d*]thiazole-2-yl)-7-chlorobenzo[*d*] oxazole-2-yl)thiophene), P5: C_18_H_9_ON_2_S_2_-Cl (Figure 1).

To study the band gap, the structural features of the polymers, we have used the DMol3 in the copyrighted Material Studio software combined with the Density Function Theory (DFT) method. To investigate band gap, the structural, calculates the thermodynamic properties of many different polymer systems [65–71], and polythiophenes [72,73]. Besides, use the GGA package [74] with takes the combined parameters of the PW91 exchange, correlation functional [75,76], and the K-point grid according to the Monkhorst–Pack diagram [77] was directed into a three-dimensional unit cell with definite dimensions a, b, c, α, β and γ as follows: P1: C_11_H_7_NS-O (a = 10 Å, b = 10 Å, c = 25 Å), P2: C_11_H_7_NS-S (a = 10 Å, b = 10 Å, c = 6 Å), P3: C_18_H_9_ON_2_S_2_-H (a = 10 Å, b = 10 Å, c = 40 Å), P4: C_18_H_9_ON_2_S_2_-OCH_3_ (a = 10 Å, b = 15 Å, c = 40 Å) and P5: C_18_H_9_ON_2_S_2_-Cl (a = 10 Å, b = 15 Å, c = 40 Å) and α = β = γ = 90°. The electrons are regarded in a homogeneous state in a system of interacting electrons through the Density Function semi-core pseudopotential [60]. During optimization of the displacement, the geometry at level 1 × 10^−5^ Ha/integer and 5 × 10^−3^ Å; and the energy was established at 1 × 10^−6^ eV. The whole process of calculating and analyzing data was based on copyrighted Material Studio software, which was set up at the Center for Computational Science of Hanoi University of Education, Vietnam.

## 3. Results and discussion

### 3.1. Effect of the material

**Figure 2.**
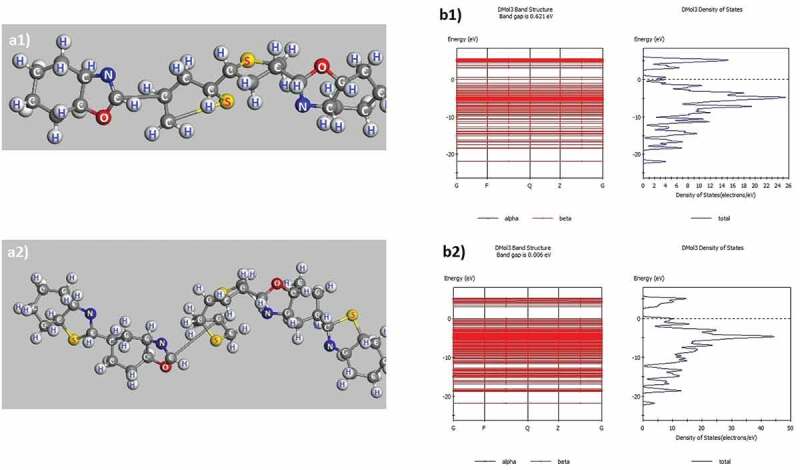
Structured shape, electronic structure of P1: C_11_H_7_NS-O (a1, b1), P3: C_18_H_9_ON_2_S_2_-H (a2, b2)

The structural features and band gap of poly C_11_H_7_NS-O (P1), C_18_H_9_ON_2_S_2_-H (P3) are shown in Figure 2 and Table 1.

**Table 1.** Structural characteristic quantities of P1 and P3

Polymer	P1: C_11_H_7_NS-O	P3: C_18_H_9_ON_2_S_2_-H
a(Å)	10.0994	10.0946
b(Å)	10.0049	9.9874
c(Å)	24.994	39.9954
α(^0^)	90.0035	89.9906
β(^0^)	90.0049	89.9875
γ(^0^)	89.9938	89.9908
E_tot_(eV)	−1904	−3348
E_g_(eV)	0.6210	0.0060

The obtained results show that in C_11_H_7_NS-O (P1) molecule, the length of C**–**H links is between 1.106 Å ÷ 1.108 Å, C**–**N is 1.459 Å ÷ 1.430 Å, C**–**S is 1.883 Å ÷ 1.924 Å, C**–**C is 1.532 Å ÷ 1.545 Å, and the link angle H**–**C**–**H is in the range of 106.260° and 106.052°, H**–**C**–**C is 109.169° ÷ 109.209°, C**–**C**–**C is 111.48° ÷ 111.457°, C**–**N**–**H is 107.658° ÷ 109.344°, C**–**S**–**C is 105.860° ÷ 105.902°, C**–**N**–**C is 86.808°, C**–**O**–**C is 107.237°. Similarly, in the C_18_H_9_ON_2_S_2_-H (P3) molecule, the C**–**H link length is 1.104 Å ÷ 1.105 Å, C**–**N is 1.419 Å ÷ 1.435 Å, C**–**S is 1.895 Å ÷ 1.991 Å, C**–**C is 1.520 Å ÷ 1.546 Å, C**–**O is 1.459 Å ÷ 1.483 Å, and link angle H**–**C**–**H is 105.910° ÷ 106.646°, H**–**C**–**C is 110.042° ÷ 110.867°, C**–**C**–**C is 108.997° ÷ 111.613°, H**–**C**–**N is 108.381° ÷ 109.471°, C**–**S**–**C is 86.276° ÷ 91.577°, C**–**N**–**C is 111.630° ÷ 112.273°. The results obtained on the link lengths are completely consistent with the previously published results **[**47**]**. Besides, the total energy of the system (E_tot_) of the P1 and P3 is E_tot_ = **–** 1904 eV and E_tot_ = – 3348 eV, respectively. When adding benzo[*d*]thiazole to the P1, the link angle and the link length of the P3 between the atoms as well as the benzene ring did not change significantly. This indicated that the structure of P1 did not change when it was added to a benzene ring although benzene ring can lead to a change in the shape, size, and total energy of the E_tot_ system (Figure 2a1, Figure 2a2) corresponding to base cell size with wide-band gap decreased from E_g_ = 0.6210 eV to E_g_ = 0.0060 eV (Table 1). These results are important bases for predicting the structure of polythiophenes containing benzo[*d*]thiazole and benzo[*d*]oxazole as well as the change of their E_tot_ and E_g_.

### 3.2. Effects of doping

**Figure 3.**
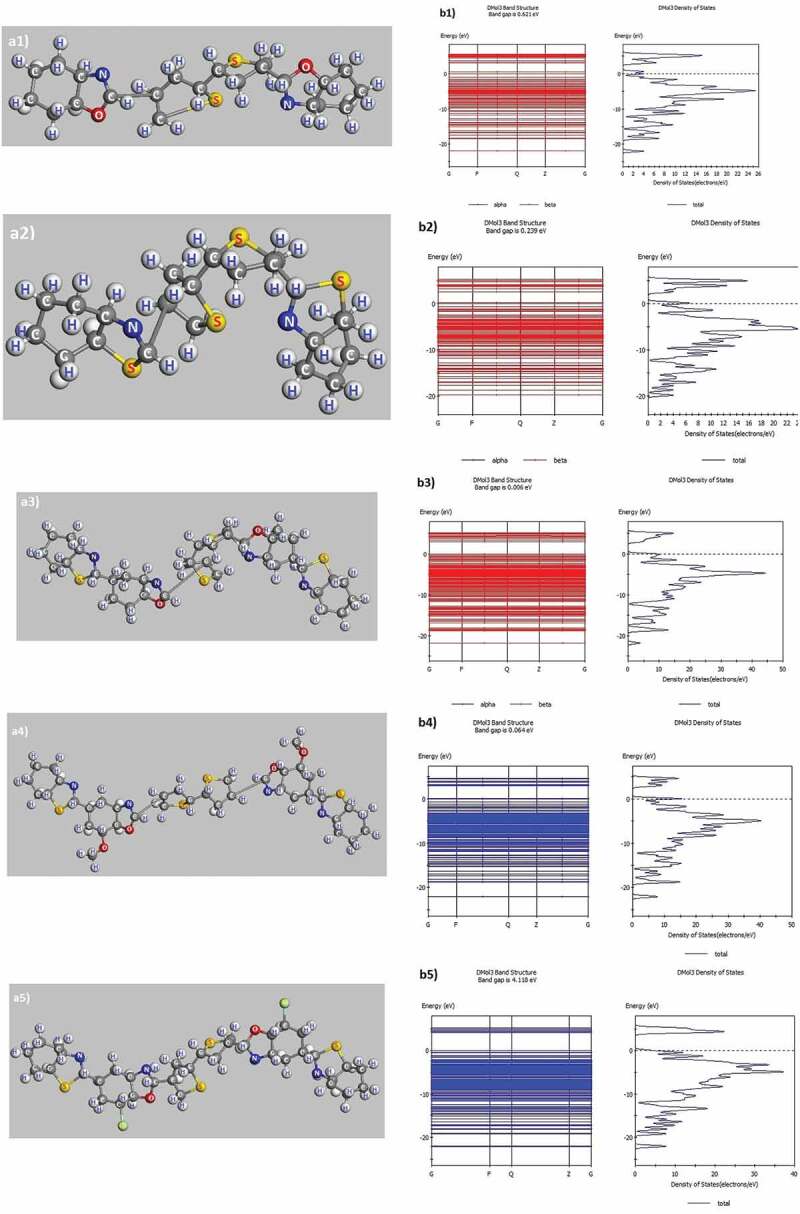
Structured shape, electronic structure of P1: C_11_H_7_NS-O (a1, b1), P2: C_11_H_7_NS-S (a2, b2), P3: C_18_H_9_ON_2_S_2_-H (a3, b3), P4: C_18_H_9_ON_2_S_2_-OCH_3_ (a4, b4), P5: C_18_H_9_ON_2_S_2_-Cl (a5, b5)

The P1 and P2 polymers were chosen as the basic materials, then their composition is changed as follows: O was substituted with S in P1: C_11_H_7_NS-O to get P2: C_11_H_7_NS-S. Similarly, replacing H with OCH_3_ in P3: C_18_H_9_ON_2_S_2_-H to get P4: C_18_H_9_ON_2_S_2_-OCH_3_ and Cl to get P5: C_18_H_9_ON_2_S_2_-Cl was done. The molecular geometry and band gap of the above samples are displayed in Figure 3 and Table 2.

**Table 2.** Structural characteristics of polymers (P1-P5) with different impurities

Polymer	P1: C_11_H_7_NS-O	P2: C_11_H_7_NS-S	P3: C_18_H_9_ON_2_S_2_-H	P4: C_18_H_9_ON_2_S_2_-OCH_3_	P5: C_18_H_9_ON_2_S_2_-Cl
a(Å)	10.0994	10.0899	10.0946	10.0976	10.0963
b(Å)	10.0049	9.9943	9.9874	15.0001	14.9870
c(Å)	24.9940	24.9972	39.9954	39.9983	39.9955
α(^0^)	90.0035	89.9978	89.9906	89.9997	89.9994
β(^0^)	90.0049	90.0006	89.9875	90.0003	90.0009
γ(^0^)	89.9938	89.9659	89.9908	89.9979	89.9997
E_tot_(eV)	–1904	–2548	–3348	–3575	–4264
E_g(_eV)	0.6210	0.2390	0.0060	0.0640	0.0645

**Figure 4.**
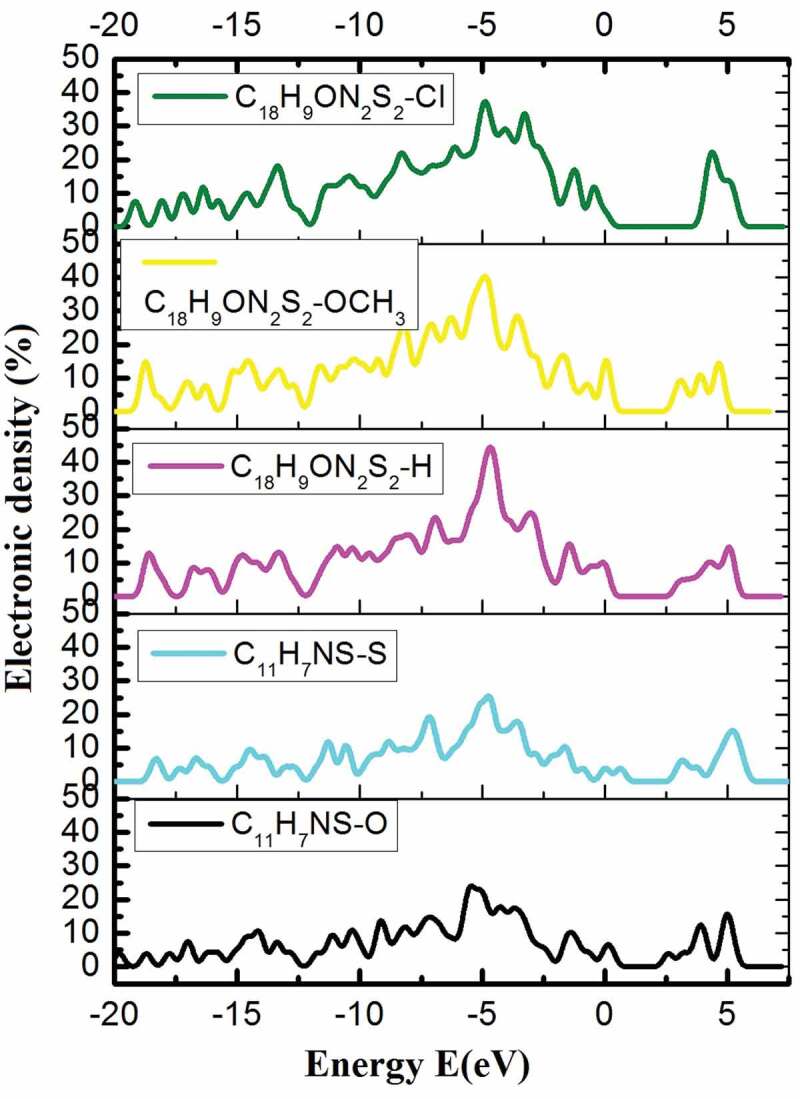
Electron density in the energy bands (E) of P1, P2, P3, P4, and P5

The network constant values such as a, b, c, α, β, and γ of the P1-P5 samples in equilibrium show that their network constant values and link angle are not changed significantly. The structural features of P1 and P2 have been reported in Figure 3a1, Figure 3b1, Figure 3a3, Figure 3b3, Table 2. Similarly, for the P_2_: C_11_H_7_NS-S has link lengths in the following range: C–H of 1.14 Å, C–N of 1.412 Å ÷ 1.448 Å, C–S of 1.880 Å ÷ 1.994 Å, C–C of 1.536 Å ÷ 1.541 Å and the bond angle between the atoms as H–C–H of 96.916° ÷ 110.185°, H–C–C of 109.135° ÷ 109.471°, C–C–C of 109.135° ÷ 113.254°, C–N–H of 108.381° ÷ 109.471°, C–S–C of 86.276° ÷ 91.577°, C–N–C of 111.630° ÷ 112.273° (Figure 3b3). For the P4: C_18_H_9_ON_2_S_2_-OCH_3_ has link lengths in the following ranges: C–H of 1.101 Å ÷ 1.103 Å, C–N of 2.709 Å, C–S of 1.452 Å ÷ 1.470 Å, C–C of 1.504 Å ÷ 1.532 Å, C–O of 1.440 Å ÷ 1.454 Å, and the following link angle between the atoms as H–C–H of 106.548° ÷ 106.711°, H–C–C of 110.285° ÷ 110.927°, C–C–C of 109.631° ÷ 109.842°, H–C–N of 109.434° ÷ 109.770°, C–S–C of 106.609° ÷ 111.975°, C–N–C of 89.924°, C–O–C of 104.513° ÷ 113.274° (Figure 3a4). For the P5: C_18_H_9_ON_2_S_2_-Cl has link lengths in the following ranges: C–H of 1.106 Å ÷ 1.107 Å, C–N of 1.444 Å ÷ 1.451 Å, C–S of 1.890 Å ÷ 1.917 Å, C–C of 1.519 Å ÷ 1.540 Å, C–O of 1.451 Å ÷ 1.507 Å, and the following link angles between the atoms as H–C–H of 106.966° ÷ 108.930°, H–C–C of 109.027° ÷ 109.918°, C–C–C of 110.002° ÷ 112.362°, H–C–N of 104.437° ÷ 106.069°, C–S–C of 105.776° ÷ 111.053°, C–N–C of 111.664°, C–O–C of 107.799°, Cl–C–C of 110.442° (Figure 3a5). The obtained results indicated that the link lengths between the atoms in the P_1_, P_2_, P_3_, P_4_, and P_5_ have values changed significantly with only the link length C–S of 1.880 Å ÷ 1.994 Å for the P1, C–O of 1.451 Å ÷ 1.492 Å for the P2, C–O of 1.459 Å ÷ 1.483 Å for the P3, C-O of 1.440 Å ÷ 1.454 Å for the P4, C–Cl of 1.873 Å ÷ 1.874 Å for the P5. The change in link lengths is due to the electrostatic interaction when the O atom in P1 is exchanged by S (in P2) and the H atom in P3 is exchanged by OCH_3_ (in P4) and Cl (in P5). This factor has led to a decrease in the total energy of the system (E_tot_) corresponding to the polymers as P1: E_tot_ of E_tot_ = – 1904 eV, P_2_: E_tot_ of E_tot_ = – 2548 eV, P3: E_tot_ of E_tot_ = – 3348 eV, P4: E_tot_ of E_tot_ = – 3575 eV, P5: E_tot_ of E_tot_ = – 4264 eV. Besides, the band gap (E_g_) corresponds to the P1: C_11_H_7_NS-O has E_g_ of E_g_ = 0.6210 eV (Figure 3b1). When replacing – O by – S, the structural shape has the form of P2: C_11_H_7_NS-S (Figure 3a2) and E_g_ of E_g_ = 0.2390 eV (Figure 3b2). Similarly, the P3, C_18_H_9_ON_2_S_2_-H has the E_g_ of E_g_ = 0.0060 eV (Figure 3b3) when replacing – H by – OCH_3_, the P4, C_18_H_9_ON_2_S_2_-OCH_3_ has the E_g_ of E_g_ = 0.0640 eV (Figure 3b4), – H equals – Cl in the P5, C_18_H_9_ON_2_S_2_-Cl with E_g_ of E_g_ = 0.0645 eV (Figure 3b5). These results indicated that when replacing O with S, the E_g_ decreased, and replacing the O atom in P1 with S led to the decrease of E_tot_ and E_g_, and with – H of P3 by – OCH_3_, – Cl, then E_g_ increased, and E_tot_ decreased. When a benzo[*d*]thiazole was added in P_1_ (changing into P_3_), the E_g_ was extremely strongly decreased, nearly 100 times (from E_g_ = 0.6210 eV to E_g_ = 0.0060 eV). The results obtained are very helpful for these future experimental researches. To confirm, we have studied the electron density in the energy bands. The results of electron density in the energy bands of C_11_H_7_NS with different impurities are shown in Figure 4.

The obtained results show that the electronic density of P1: C_11_H_7_NS-O, P3: C_18_H_9_ON_2_S_2_-H with the energy bands (E) of E = – 20 eV, – 15 eV, – 10 eV, – 7.5 eV, – 5 eV, 0.00 eV, 5 eV, or 7.5 eV has the corresponding electronic density P1: 0.00%, 4.55%, 2.69%, 18.76%, 23.66%, 3.96%, 13.80%, 0.00%; P3: 0.00%, 10.98%, 11.57%, 17.29%, 36.84%, 9.71%, 14.45%, 0.00%. When doping the functional groups – S, – OCH_3_, or – Cl into P1 and P3, we obtained P2, P4, and P5, which showed significant changes in electron density. For example, in the E = – 20 eV energy range, the electron density has increased from 0.00% to 2.55% then back to 0.00%; in the energy range of E = – 15 eV, the electron density decreases and then increases and vice versa from 4.55% to 4.41%, up 10.98%, 11.82%, down to 6.26%; in the energy range of E = – 10 eV, the electron density increases and then decreases from 2.69% to 7.34%, 11.57%, 14.58%, down to 12.05%; at the energy range of – 7.25 eV, the electron density change to reach the extreme value in the valence area, tends to change from 18.76% to 14.57% to 17.29%, 22.13%, to 17.21%; in the E = −5 eV energy range, the density of electrocytes change from 23.66% to 22.27% to 36.84%, 39.75%, 36.15%; in the range with E = 0.00 eV the electronic density increased and then decreased from 3.96% to 5.80%, 9.71%, 14.94%, 4.83%; in the energy range of E = 5 eV, the electron density increased and then decreased from 13.80% to 15.62%, down to 14.45%, 3.55% to 13.80%; in the energy range E = 7.25 eV, the polymer has an electron density of 0.00%. This showed that in the electron density, the valence region had the maximum value, the largest percentage in the energy range of E = – 5 eV (Figure 4). Through the obtained calculations, we confirm that this is still a semiconductor material and conductivity was increased because doping the -S functional group into P1 leads to band gap E_g_ decrease and the conductivity increase. Similarly, doping the functional groups – OCH_3_, or – Cl into P3 leads to an increase in the band gap of E_g_. Besides, connecting P_1_ and P_3_ through the benzene bridge results in a huge decrease of E_g_ nearly 100 times, while E_tot_ decreased nearly 2 times. The results are shown in the first Brillouin region (corresponding to zero) correspond to an increase in electron density from 3.96% to 5.80%, 9.71%, 14.94%, and then to 4.83% that increase in electrical mobility. The electrical mobility of P_1_ decreased when it was doped with -S, and increased when – OCH_3_ or – Cl was added to P3. It confirmed that the impurities affected the network structure and electronic structure of P_1_ and P_3_. The cause of this phenomenon is due to the influence of the electronic structure of the functional groups on the band gap E_g_ and the total energy of the system E_tot_. The obtained results are very useful for future experimental results used to fabricate smart electronic device.

## 4. Conclusions

This paper summarizes the structural calculations and gives a detailed comparison of the lattice structure, and electronic structure of C_11_H_7_NS-X and C_18_H_9_ON_2_S_2_-R in case of X and R was replaced with other elements or groups. The change in link length, link angle, total energy, and band gap of doped polythiophenes is convincing evidence to confirm the influence of the impurities, the benzene ring on the band gap, and electronic structure features of polymers. Among investigated dopants, the benzo[*d*]thiazole ring exhibits the strongest effect on the band gap polymers. These results are considered the basis for future experimental work related to doping on the polythiophenes containing benzo[*d*]thiazole and benzo[*d*]oxazole and used to fabricate smart electronic device.

## Data Availability

The data that support the findings of this study are available from the corresponding author upon reasonable request.
